# Correction: Titmuss et al. Immune Activation Following Irbesartan Treatment in a Colorectal Cancer Patient: A Case Study. *Int. J. Mol. Sci.* 2023, *24*, 5869

**DOI:** 10.3390/ijms252212225

**Published:** 2024-11-14

**Authors:** E. Titmuss, K. Milne, M. R. Jones, T. Ng, J. T. Topham, S. D. Brown, D. F. Schaeffer, S. Kalloger, D. Wilson, R. D. Corbett, L. M. Williamson, K. Mungall, A. J. Mungall, R. A. Holt, B. H. Nelson, S. J. M. Jones, J. Laskin, H. J. Lim, M. A. Marra

**Affiliations:** 1 Canada’s Michael Smith Genome Sciences Centre, BC Cancer, Vancouver, BC V5Z 4S6, Canada; 2 Deeley Research Centre, BC Cancer, Victoria, BC V8R 6V5, Canada; 3 Department of Pathology and Laboratory Medicine, University of British Columbia, Vancouver, BC V6T 1Z7, Canada; 4 Pancreas Centre BC, Vancouver, BC V5Z 1G1, Canada; 5 Department of Medical Oncology, BC Cancer, Vancouver, BC V5Z 4E6, Canada; 6 Department of Medical Genetics, University of British Columbia, Vancouver, BC V6T 1Z2, Canada

The journal’s Editorial Office and Editorial Board are jointly issuing a resolution and removal of the *Journal Notice* linked to this article [1]. Following concerns raised about the integrity of the peer-review (misconduct by one of two reviewers), the Editorial Office has conducted a post-publication peer-review of this article. This process included the recruitment of a new independent reviewer and was supervised by the original Academic Editor to ensure full compliance with MDPI’s Editorial Process (https://www.mdpi.com/editorial_process). The authors were unaware of misconduct by the reviewer, and had not included any suggestions by the reviewer for inappropriate references.

As a result of this process, the Academic Editor and the authors have agreed to update the following aspect(s) of this publication:

Reviewer report 2 has been removed from the peer-review record, while one new review report has been added and uploaded to the peer-review record (https://www.mdpi.com/1422-0067/24/6/5869/review_report).

With this update, the Academic Editor is satisfied that the editorial process related to this article has been completed as per MDPI’s Editorial Process policy. The Editorial Office would like to thank the authors for their collaboration during this process.
