# Supplementary figures and images for: Immune Activation Following Irbesartan Treatment in a Colorectal Cancer Patient: A Case Study
Source: Int J Mol Sci. 2023 Mar 20;24(6):5869. doi: 10.3390/ijms24065869 (PMC10051648; doi:10.3390/ijms24065869)

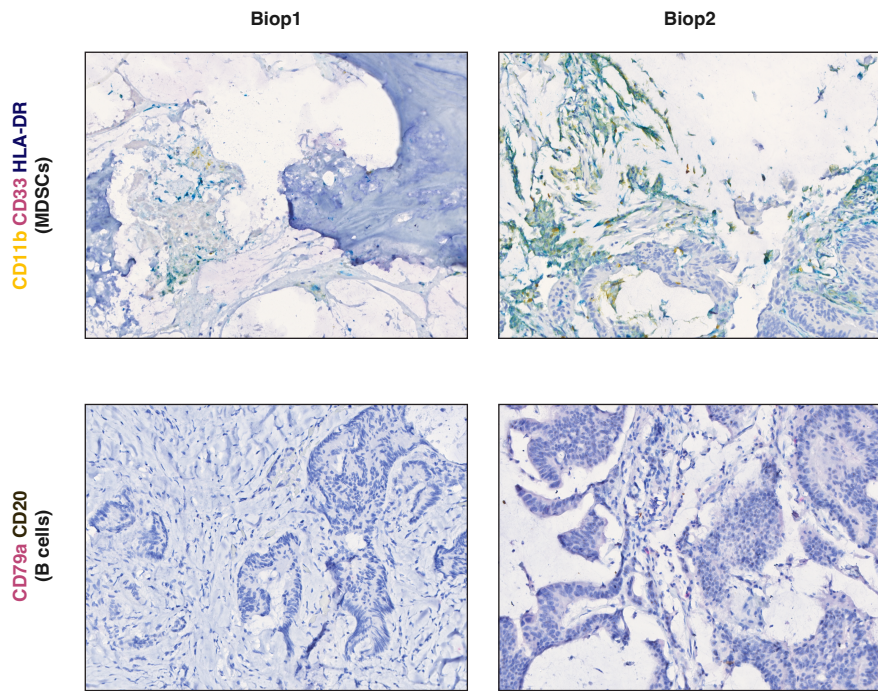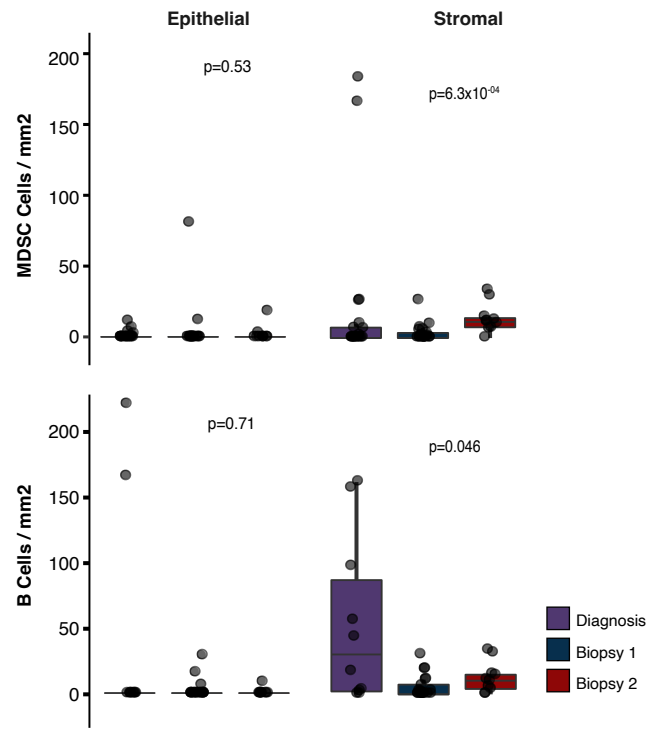

Supplement: Supplementary file 1 [file ijms-24-05869-s001.zip › Supplemental Figure S1.pdf]
